# Association Between Single-Nucleotide Polymorphisms and DRAM1 Gene Expression in Periodontal Ligament Fibroblasts Under Orthodontic Compression

**DOI:** 10.3390/biomedicines14071421

**Published:** 2026-06-23

**Authors:** Rebecca Linke, Erika Calvano Küchler, Peter Proff, Christian Kirschneck, Agnes Schröder, Svenja Beisel-Memmert

**Affiliations:** 1 Department of Orthodontics, Medical Faculty, University of Bonn, 53111 Bonn, Germany; 2 Department of Orthodontics, Medical Faculty, University of Regensburg, 93047 Regensburg, Germany; 3 Department of Orthodontics, Center for Dentistry and Oral Medicine (Carolinum), Goethe University Frankfurt am Main, 60596 Frankfurt am Main, Germany

**Keywords:** gene, autophagy, periodontal ligament fibroblast, DNA damage-regulated autophagy modulator 1, orthodontic tooth movement, single nucleotide polymorphisms

## Abstract

**Background/Objectives**: Autophagy is a key degradative pathway involved in orthodontic tooth movement. DNA damage-regulated autophagy modulator 1 (DRAM1), a protein that plays a central role in the degradation of autophagic cargo, exhibits differential regulation in human periodontal ligament (hPDL) fibroblasts under compressive force. Single-nucleotide polymorphisms (SNPs) may influence force-induced gene expression. Therefore, this study investigated the impact of DRAM1 SNPs on its expression in hPDL fibroblasts under compression force. **Methods**: The hPDL sample comprised cells of 59 patients. A physiological compressive strain of 2 g/cm^3^ was used to simulate orthodontic tooth movement. Total RNA from hPDL fibroblasts was isolated to determine DRAM1 relative gene expression under loaded conditions and in a physiological control. Furthermore, a genotyping analysis of six SNPs within the *DRAM1* gene (rs756534 (G/T), rs2138257 (C/T), rs2176092 (C/T), rs4622329 (A/G), rs10860812 (A/G), and rs4764657 (A/G)) was performed using real-time polymerase chain reaction. *DRAM1* expression was com-pared among genotypes of each SNP using an alpha of 5%. Linear regression analysis was then employed to evaluate SNP-SNP interaction. **Results**: The relative *DRAM1* gene expression was not statistically significantly different (*p* > 0.05) according to the geno-types. The SNP-SNP interaction did not demonstrate any statistically significant associ-ation either. **Conclusions**: *DRAM1* gene expression in hPDL fibroblasts under orthodontic compression may not be regulated by the studied intronic SNPs in the gene encoding DRAM1.

## 1. Introduction

The aim of orthodontics is to restore functional balance to the masticatory system by guiding teeth into physiologically optimal alignment within the anatomical boundaries of the alveolar bone. The central biological process of orthodontics is orthodontic tooth movement (OTM). Tooth movement depends on several patient- and treatment-related factors, including the patient’s age and health status, the magnitude of the applied mechanical forces and the duration of treatment [1,2]. Mechanical forces trigger highly regulated cellular and molecular responses in the periodontal ligament (PDL) and the surrounding alveolar bone, ultimately enabling stable and functional occlusion [3]. The PDL is a specialized connective tissue that surrounds the roots of teeth and anchors them to the alveolar bone [4]. During orthodontic treatment, applied mechanical forces generate areas of compression and tension within the PDL, leading to alterations in the activity of resident cells. The predominant cell population within the PDL consists of fibroblast-like cells, which function as mechanosensitive cells capable of detecting and responding to mechanical stress. In response to force application, these cells release a variety of signaling molecules, including inflammatory cytokines, prostaglandins and growth factors, which initiate and regulate the process of alveolar bone remodeling [5]. A well-known key regulatory pathway involved in this process is the receptor activator of nuclear factor kappa-B ligand (RANKL)/osteoprotegerin (OPG) system [6]. Increased expression of RANKL promotes osteoclast differentiation and activation through its interaction with the receptor activator of nuclear factor kappa-B (RANK), thereby stimulating bone resorption on the compression side. In contrast, OPG acts as a decoy receptor that binds RANKL and inhibits osteoclastogenesis, thereby modulating the extent of bone resorption. Concurrently, osteoblast-mediated bone formation predominates on the tension side. The coordinated balance between these catabolic and anabolic processes controls tooth movement.

While the application of physiological stress is needed for tissue homeostasis, prolonged mechanical stress or mechanical overload can lead to cell death and compromised tissue integrity [4,5,7]. One of the key cellular mechanisms which are important for mechanical stress adaptation is autophagy. Autophagy involves the conserved degradation of unnecessary or dysfunctional cellular components to sustain cellular homeostasis [8]. It is an adaptive process that, depending on the stress level, can promote cell survival or cell death [9]. During OTM, autophagy has been shown to promote tissue remodeling, reduce inflammation and be crucial for the adaptation of PDL fibroblasts to mechanical forces [10,11,12,13], supporting their survival and function, particularly under the influence of continuous mechanical stress. However, autophagy is a complex process that involves many molecules, and the underlying cellular mechanisms in OTM still need to be investigated. One noteworthy protein in this process is damage-regulated autophagy modulator 1 (DRAM1). DRAM1 is a lysosomal membrane protein required to conclude autophago-somal degradation by mediating the interaction between the autophagosome and lysoome [14]. As a target protein of p53, DRAM1 is additionally involved in the p53-dependent apoptotic cell death [15,16,17]. DRAM1 has already been shown to play a pivotal role in the adaptation of PDL fibroblasts to inflammatory and microbial challenges [18]. Furthermore, a recent study described that DRAM1 expression is upregulated in PDL fibroblasts under mechanical stress [19], suggesting that DRAM1-induced autophagy may support PDL fibroblast adaptation to the mechanical forces during OTM.

The gene encoding DRAM1 is located on chromosome 12q23.2, spans about 134.5 kb and is composed of eight coding exons. For the DRAM1 gene, several genetic polymorphisms are known, such as single-nucleotide polymorphisms (SNPs) that have been studied in different health conditions and are associated with Tourette syndrome, systemic lupus erythematosus and responses in non-small cell lung cancer [20,21,22]. SNPs are DNA variations in a single nucleotide that differ among biological species or paired chromosomes in an individual. Previous studies reported that SNPs in several genes associated with OTM affect the expression of those genes in PDL fibroblasts under mechanical forces and that fibroblasts with specific phenotypes respond differently to the mechanical stress [23]. Genetic association studies have demonstrated that specific SNPs may function as critical biomarkers for an individual patient’s response to orthodontic treatment, including risk-increasing variants in inflammatory pathways linked to orthodontically induced external apical root resorption [24,25], as well as the association with an increased susceptibility to dental pulp calcification [26] following the application of orthodontic forces.

To our knowledge, this is the first study to investigate the association of SNPs within the *DRAM1* gene with PDL fibroblast adaptation under orthodontic compressive force, thereby providing novel insight into the genetic regulation of orthodontic tissue remodeling. We hypothesized that intronic SNPs in *DRAM1* are involved in a differential upregulation of its expression as a response to compressive forces, by affecting transcription factor binding or modulating chromatin structure. Therefore, in this in vitro study, we investigated the effect of the intronic SNPs rs756534, rs2138257, rs2176092, rs4622329, rs10860812 and rs4764657 in the *DRAM1* gene on its expression pattern in human PDL fibroblasts under mechanical forces.

## 2. Materials and Methods

### 2.1. Ethics

Sample collection and experimental procedure was approved by the ethics committee of the University of Regensburg, Germany (Approval No. 12-170-0150).

### 2.2. Sample Collection and In Vitro Compression Model for Orthodontic Tooth Movement

**Figure 1:**
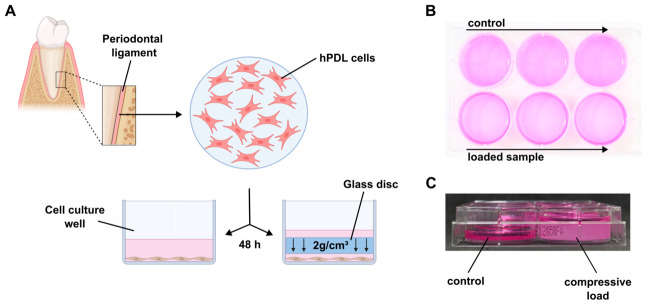
Experimental design. (**A**) Human periodontal ligament (hPDL) cells were isolated from the periodontal tissue of extracted healthy teeth and cultured under growth-stimulating conditions. Orthodontic compressive force of 2 g/cm^2^ was simulated by applying a 17.1 g glass disc to the well (**right side**) for 48 h. As physiological control, no pressure was applied (**left side**). (**B**,**C**) Representative images of the experimental mechanical loading setup showing loaded samples exposed to a compressive force (2 g/cm^3^) and unloaded control samples in 6-well cell culture plates.

Human periodontal ligament (hPDL) samples from 59 patients (28 males and 31 females; age: 13–31 years) undergoing dental treatment at the maxillofacial surgery clinic at the University of Regensburg (Germany) were collected from carries-free third molars with no sign of periodontal disease extracted in a routine dental treatment. If the patient had more than one tooth extracted, only one tooth per patient was selected. The hPDL was isolated and cultivated, and hPDL fibroblasts were characterized according to a well-established protocol as previously described [27,28,29]. Briefly, isolated hPDL tissue samples were cultured in 6-well plates in DMEM high-glucose medium (Gibco^TM^, Thermo Fisher Scientific, Waltham, MA, USA) supplemented with 10% FCS (P30–3306, PAN-Biotech, Aidenbach, Germany), 1% L-glutamine (SH30034.01, GE-Healthcare-Europe, Munich, Germany), 100 µM ascorbic acid (A8960, Sigma-Aldrich^®^, St. Louis, MO, USA), and 1% antibiotics/antimycotics (A5955, Sigma-Aldrich^®^) under normal cell culture conditions (37 °C, 5% CO_2_). Following proliferation, adherent hPDL fibroblasts, which were identified based on their morphological spindle shape, were frozen in liquid nitrogen (90% FCS, 10% DMSO, freezing 1 °C/min) and stored until further use. For in vitro experiments, pooled hPDL fibroblasts of the fifth passage were randomly seeded at a density of 2000 cells/mm^2^ per 6-well plate. A physiological compressive strain (orthodontic force) of 2 g/cm^2^ was applied for 48 h according to Kanzaki et al. (2002) by using a sterile glass disc to simulate orthodontic tooth movement in pressure areas of the hPDL [30]. This was performed under cell culture conditions at 70% confluency without changing the medium, according to published protocols [27,28,29]. The scheme of the in vitro experiment is shown in Figure 1.

### 2.3. Total RNA Isolation and Quantification of Relative Gene Expression (RT-qPCR)

Total RNA from hPDL fibroblasts was isolated using peqGOLD TriFastTM (PEQLAB Biotechnology GmbH, Erlangen, Germany) following manufacturer’s instructions. The RNA was instantly cooled on ice. RNA quantity and purity were photometrically determined at 280 nm, 260 nm, and 230 nm (NanoPhotometer N60, Implen, Munich, Germany). For complementary DNA (cDNA) synthesis, 500 ng RNA per sample were transcribed using a Mastercycler ep realplex-S thermocycler (Eppendorf AG, Hamburg, Germany). The relative expression of DRAM1 was determined by RT-qPCR in a StepOnePlus thermocycler (Thermo Fisher Scientific, Waltham, MA, USA) using 1.5 µL cDNA per sample, the TaqManTM Fast Advanced Mastermix (#44444556, Applied Biosystems^TM^, Thermo Fisher Scientific, Waltham, MA, USA) and the primer pair Hs01022842_m1 (Thermo Fisher Scientific, Waltham, MA, USA) according to manufacturer’s instructions. For normalization of the target gene, the two previously established reference genes RPL22 and PPIB were used [28]. A non-template control without cDNA was tested to evaluate primer dimers or contaminating DNA. The relative gene expression of DRAM1 was calculated by 2^−ΔCq^ with ΔCq = Cq (DRAM1) − Cq (mean RPL22/PPIB), divided by the respective arithmetic 2^−ΔCq^ mean of the untreated samples.

### 2.4. DNA Extraction and Genotyping Analysis

The DNA of hPDL cells was isolated using the GenElute Mammalian Genomic DNA Miniprep kit (Sigma Aldrich, Munich, Germany) according to the manufacturer’s instructions. DNA quantification and purity were photometrically determined at 260 nm and 230 nm (NanoPhotometer N60, Implen, Munich, Germany). A total of six SNPs in the DRAM1 gene, which is located in 12q23.2, were selected based on their minor allele frequency (>10%). The SNPs’ characteristics are presented in Table 1. Genotyping was performed by allelic discrimination real-time PCR using the TaqMan assay in the StepOnePlus thermocycler (Thermo Fisher Scientific, Waltham, MA, USA) as previously described [23].

### 2.5. Statistical Analysis

Statistical analyses were performed using the software GraphPad Prism 10.2.3 (GraphPad Software, Inc., San Diego, CA, USA). The Shapiro–Wilk test was used to assess the normality of the gene expression data, while Levene’s test was used to determine the homogeneity of variance across groups. A non-parametric Kruskal–Wallis test was used to evaluate significant differences between three or more groups. Kruskal–Wallis with Dunn’s multiple comparison test was used to compare DRAM1 expression levels among the three genotypes of each SNP in the co-dominant model. Dunn’s test was used as the post hoc test. Linear regression analysis was performed to evaluate SNP–SNP interaction. Statistical significance was established at an alpha level of 5% (*p* < 0.05) for all analyses.

## 3. Results

The SNPs investigated in this study are all intronic variants. The genotyping success rate (success of PCR amplification) of each SNP was higher than 95% (Table 2). The de-viation of the genotype frequencies in the population from the expectations of the Hardy–Weinberg (HW) equilibrium was examined using the Chi-square distribution. The Chi-square distribution represents the theoretical distribution of sample values under HW equilibrium. Since Χ2 < 3.841 and *p* > 0.05, we conclude that the genotype frequencies in this population are not significantly different from what would be expected if the population was in HW equilibrium.

In order to determine whether *DRAM1* gene expression is regulated by any of the six investigated SNPs evaluated in the *DRAM1* gene (rs756534, rs2138257, rs2176092, rs4622329, rs10860812, and rs4764657), the relative expression of *DRAM1* was compared to the genotypes of each SNP.

The relative *DRAM1* expression was not significantly different (*p* > 0.05) according to the genotypes of the six SNPs (Table 3), indicating that the studied SNPs in the *DRAM1* gene are not associated with *DRAM1* gene expression.

To identify SNP-SNP interactions, linear regression analysis was performed. The regression analysis did not show any statistically significant difference, demonstrating that there is no association between the SNPs (Table 4).

## 4. Discussion

OTM describes a process in which mechanical forces are applied to the periodon-tium of malpositioned teeth, resulting in alveolar bone remodeling. In recent years, the molecular mechanisms underlying OTM have increasingly come into focus. The over-arching aim is to identify the key regulatory factors involved and, based on this knowledge, to develop therapeutic strategies that accelerate tooth movement while minimizing adverse effects such as root resorption [27,28,29]. It is well established that PDL fibroblasts from different individuals respond heterogeneously to mechanical loading, indicating inter-individual variability in the gene regulatory response to mechanical stress [23,31,32]. Among the processes critically involved in OTM is autophagy, as convincingly demonstrated by animal studies using pharmacological modulation of autophagy and by in vitro experiments subjecting human PDL fibroblasts to mechanical strain [11,12,33,34]. Of particular interest in this context is the gene *DRAM1* [19]. *DRAM1* encodes a lysosomal membrane protein that plays a pivotal role in autophagy by facilitating the interaction between autophagosomes and lysosomes [14,15]. In addition, as a transcriptional target of p53, DRAM1 contributes to p53-mediated apoptosis [16,17]. This dual role positions DRAM1 as a potential molecular switch between cytoprotective autophagy under physiological loading and autophagy-induced cell death under overload conditions [19]. Consistent with this notion, former findings suggest that DRAM1 is essential for the adaptation of PDL fibroblasts to both inflammatory and microbial challenges [18].

In recent decades, the number of studies associating SNPs with orthodontic phenotypes in dental patients has been increasing [24,25,26,35,36,37,38]. Some SNPs have even been pointed out as potential markers to predict inflammatory root resorption induced by orthodontic treatment [24,25]. In vitro studies have also explored the role of SNPs in orthodontics [23,31,32]. A study by Küchler et al. (2021) focused on the role of vitamin D during the response to OTM [32]. The study reported that PDL fibroblasts from individuals carrying the GG genotype in the SNP rs739837 in the gene encoding vitamin D receptor (VDR) presented lower VDR mRNA expression and those from individuals carrying the CC genotype in the SNP rs7975232 presented higher VDR mRNA expression under mechanical forces. Cells obtained from individuals carrying the GG genotype in rs739837 presented lower VDR mRNA expression and those from individuals carrying the CC genotype in rs7975232 presented higher VDR mRNA expression [32]. Since *DRAM1* expression is upregulated in these cells following force application, it is worthy investigating whether SNPs in the gene encoding DRAM1 affect its expression during OTM in PDL fibroblasts.

Multiple SNPs within the *DRAM1* gene have been identified and investigated in some health conditions so far [20,21,22]. In this study, we examined six SNPs in the *DRAM1* gene (rs756534, rs2138257, rs2176092, rs4622329, rs10860812, and rs4764657) that were selected based on their minor allele frequency. In a case–control cohort from southern China, rs4622329 was shown to genetically interact with the SNP rs7574865 in STAT4 and that the interaction of these SNPs is associated with biliary atresia [39]. However, the underlying mechanisms of genetic interactions and biliary atresia association need to be further explored. Moreover, Song et al. suggested a possible role of rs4622329 in the context of lupus nephritis [40]. In a lupus nephritis genome-wide association study (GWAS), rs4622329 was identified as one out of nine SNPs showing *p* < 0.5 and one out of three SNPs with a consistent odds ratio. The SNP rs10860812 was reported to be associated with hypertension. However, a secondary follow-up study in a larger cohort failed to reveal the association with blood pressure, although the results pointed towards a protective effect for the A-allele [41]. For the other SNPs investigated in our study, no study results or associations with diseases are known so far. The functional mechanisms of these SNPs are widely unknown. However, due to their intronic location, we suggested that these non-coding SNPs may boost gene expression by affecting regulatory elements, such as transcription factor binding sites or by changing the chromatin structure through epigenetic modifications.

In our study, the examined SNPs were not associated with the relative *DRAM1* gene expression under physiological compressive force, suggesting that these SNPs are not involved in a differential regulation of *DRAM1* as a reaction to pressure. The tested SNPs may have post-transcriptional rather than transcriptional regulatory effects, affecting RNA splicing or mRNA stability. However, this was beyond the scope of this study. Future studies should investigate post-transcriptional or epigenetic regulation mechanisms, including miRNAs, DNA methylation or chromatin remodeling, which may influence *DRAM1* expression.

Nevertheless, based on our findings, this study indicates that the tested SNPs within the *DRAM1* gene may not serve as biomarkers for an individual patient’s response to orthodontic treatment.

The study has some important limitations that should be discussed. One limitation is that not all SNPs present within the *DRAM1* gene were investigated here due to their minor allele frequency. Furthermore, another limitation is the limited sample size. We evaluated samples from only 59 patients. Hence, future studies should include a larger sample size from different ethnicities and the investigation of more SNPs to cover the *DRAM1* gene to validate the role of SNPs on *DRAM1* expression. Additionally, in this study we only investigated the effect of SNPs on *DRAM1* gene expression under mechanical load. However, Mannes et al. showed that *DRAM1* expression was significantly upregulated under pathological overload after 24 h [19]. Nevertheless, mechanical overload resulting in upregulated regulation could also lead to dispersion within the data. Hence, we decided to test the effect of mechanical load instead of overload, since this force is the most relevant one for OTM in clinics.

To date, only little is known about genetic factors affecting gene expression during OTM. Therefore, the identification of SNPs impacting orthodontic treatment should prospectively receive more attention.

## 5. Conclusions

Knowledge of genetic markers that can predict slow and fast movers in orthodontic practice will help to develop personalized treatment plans for patients. In our study, we investigated the impact of six SNPs (rs756534, rs2138257, rs2176092, rs4622329, rs10860812 and rs4764657) on the regulation of *DRAM1* gene expression in the context of orthodontic tooth movement. The tested SNPs did not show a significant association with *DRAM1* gene expression in hPDL fibroblasts under physiological compressive force, revealing that those SNPs within the *DRAM1* gene may not serve as biomarkers for an individual patient’s response to orthodontic treatment.

## Figures and Tables

**Table 1:** *DRAM1* SNP characteristics.

SNP ID	Base Change #;*	Minor Allele Frequency
rs756534	**G**/T	0.42
rs2138257	**C**/T	0.41
rs2176092	**C**/T	0.44
rs4622329	**A**/G	0.40
rs10860812	**A**/G	0.45
rs4764657	A/**G**	0.47

# https://www.ncbi.nlm.nih.gov/snp (accessed on 25 July 2025) * Bold forms indicate the minor allele.

**Table 2:** Genotyping success rate and Hardy-Weinberg equilibrium analysis of each SNP.

SNP ID	Genotyping Rate	Χ2 *	*p*-Value *
rs756534	58/59 (98.3%)	2.68	0.10
rs2138257	57/59 (96.6%)	0.47	0.49
rs2176092	58/59 (98.3%)	0.93	0.33
rs4622329	58/59 (98.3%)	1.39	0.24
rs10860812	59/59 (100%)	1.8	0.18
rs4764657	59/59 (100%)	1.66	0.19

* Values for Hardy-Weinberg equilibrium.

**Table 3:** DRAM1 expression according to the genotypes.

SNP ID	Genotype	*n*	Fold Change DRAM-1 Expression			*p*-Value
			Median	25th Percentile	75th Percentile	
rs756534	GG	6	1.37	0.98	2.08	0.589
	GT	30	1.21	1.00	1.34	
	TT	14	1.19	0.97	1.69	
rs2138257	CC	7	1.15	0.98	1.82	0.985
	CT	26	1.23	1.04	1.56	
	TT	16	1.26	0.91	1.43	
rs2176092	CC	8	1.15	0.96	1.67	
	CT	28	1.23	1.06	1.50	0.743
	TT	14	1.28	0.89	1.55	
rs4622329	AA	6	1.17	0.92	1.39	
	AG	28	1.23	1.01	1.50	0.749
	GG	16	1.22	0.95	1.79	
rs10860812	AA	8	1.12	0.96	1.67	
	AG	30	1.24	1.06	1.45	0.717
	GG	13	1.27	0.89	1.67	
rs4764657	AA	12	1.19	1.01	1.78	
	AG	30	1.23	1.04	1.34	0.863
	GG	9	1.31	0.93	1.67	

**Table 4:** Linear regression analysis to evaluate SNP-SNP interaction.

Variable	Reference	Estimate	Standard Error	95% CI	t	*p*-Value
rs756504 [TT]	GT	0.09	0.15	−0.21 to 0.39	0.61	0.547
rs756504 [GG]		0.28	0.20	−0.13 to 0.69	1.36	0.179
rs2108257 [CT]	TT	0.00	0.15	−0.30 to 0.31	0.02	0.980
rs2108257 [CC]		0.00	0.21	−0.43 to 0.43	0.01	0.989
rs2176092 [CT]	TT	−0.02	0.15	−0.33 to 0.29	0.12	0.906
rs2176092 [CC]		−0.08	0.21	−0.50 to 0.33	0.40	0.692
rs4622029 [AG]	GG	−0.05	0.15	−0.34 to 0.24	0.34	0.734
rs4622029 [AA]		−0.15	0.22	−0.59 to 0.30	0.66	0.513
rs10860812 [AG]	GG	−0.02	0.15	−0.33 to 0.29	0.12	0.906
rs10860812 [AA]		−0.09	0.21	−0.50 to 0.33	0.41	0.683
rs4764657 [AA]	AG	0.13	0.16	−0.18 to 0.45	0.83	0.408
rs4764657 [GG]		0.13	0.17	−0.22 to 0.48	0.75	0.460

## Data Availability

Data are contained within the article.

## Abbreviations

The following abbreviations are used in this manuscript:

ATG	Autophagy-related
DRAM1	DNA damage-regulated autophagy modulator 1
GWAS	Genome-wide association study
hPDL	Human periodontal ligament
OTM	Orthodontic tooth movement
PDL	Periodontal ligament
SNP	Single-nucleotide polymorphism
